# A streamlined method for the fast and cost-effective detection of bacterial pathogens from positive blood cultures for the BacT/ALERT blood culture system using the Vitek MS mass spectrometer

**DOI:** 10.1371/journal.pone.0267669

**Published:** 2022-04-28

**Authors:** Johannes Forster, Britta Kohlmorgen, Julian Haas, Philipp Weis, Lukas Breunig, Doris Turnwald, Boris Mizaikoff, Christoph Schoen

**Affiliations:** 1 Institute for Hygiene and Microbiology, University of Würzburg, Würzburg, Germany; 2 Institute of Hygiene and Environmental Medicine, Charité – Universitätsmedizin Berlin, Berlin, Germany; 3 Hahn-Schickard- Society for Applied Research, Ulm, Germany; 4 Department of Internal Medicine I, Caritas-Krankenhaus Bad Mergentheim, Bad Mergentheim, Germany; 5 Department of Internal Medicine – Cardiology, DRK Klinikum Berlin Westend, Berlin, Germany; 6 Institute of Analytical and Bioanalytical Chemistry, Ulm University, Ulm, Germany; Fisheries and Oceans Canada, CANADA

## Abstract

**Background and objective:**

Prompt pathogen identification of blood stream infections is essential to provide appropriate antibiotic treatment. Therefore, the objective of this prospective single centre study was to establish an inexpensive, fast and accurate protocol for bacterial species identification with SDS protein-extraction directly from BacT/Alert^®^ blood culture (BC) bottles by VitekMS^®^.

**Results:**

Correct species identification was obtained for 198/266 (74.4%, 95%-CI = [68.8%, 79.6%]) of pathogens. The protocol was more successful in identifying 87/96 (91.4%, 95%-CI = [83.8%, 93.2%]) gram-negative bacteria than 110/167 (65.9%, 95%-CI = [58.1%, 73.0%]) gram-positive bacteria. The hands-on time for sample preparation and measurement was about 15 min for up to five samples. This is shorter than for most other protocols using a similar lysis-centrifugation approach for the combination of BacT/Alert^®^ BC bottles and the Vitek^®^ MS mass spectrometer. The estimated costs per sample were approx. 1.80€ which is much cheaper than for commercial kits.

**Conclusion:**

This optimized protocol allows for accurate identification of bacteria directly from blood culture bottles for laboratories equipped with BacT/Alert^®^ blood culture bottles and VitekMS^®^ mass spectrometer.

## Introduction

Bloodstream infections (BSI) are one of the leading causes of death worldwide with fatality rates being as high as one fifth of patients [1,2]. Fast identification of the causative pathogen is essential to select appropriate treatment [3]. Therefore, rapid diagnostic test (RDT) may have positive impacts on appropriate antibiotic treatment, clinical outcomes and health care costs [4–7].

Different DNA-based methods are commercially available to shorten the time to identify pathogens in positive blood cultures (BCs) [8,9] but are expensive in acquisition and/or expendables. Due to its accuracy, very short time-to-result and low running costs, mass spectrometry (MS) became the standard method of bacterial identification in many clinical laboratories [10]. While different commercial mass spectrometers are in use, BioTyper^®^ (Bruker Daltonics, Bremen, Germany) and Vitek MS^®^ (bioMérieux, Marcy l’Étoile, France) are the most common systems for clinical use worldwide.

To date, various groups reported successful identification of bacteria directly from positive BCs using MS and commercial preparation kits for both systems (Sepsityper Kit, Bruker Daltonics, and Vitek^®^ MS Blood Culture Kit, bioMérieux) are available. In addition, numerous in-house protocols with non-inferior or improved performance have been established. While separation and extraction method vary, most of the protocols have in common that lysis of blood cells is followed by centrifugation (lysis-centrifugation method) or filtration (lysis-filtration method) and several washing steps [11–16].

The effect on the results of RDT of the blood culture bottles widely used (Bact/ALERT^®^ [bioMérieux] and BD BACTEC [BectonDickinson, Franklin Lakes, NJ, USA]) been studied and discussed previously [17] Most data on the performance of different RDT protocols is available for the combination of BD BACTEC (BectonDickinson) BC bottles and BioTyper^®^ MS (Bruker) [18].

In order to have an impact on routine clinical management a RDT for the direct identification of bacteria from positive BCs has to be cheap, fast, reproducible, accurate, without any specialized equipment needed and easy to integrate into the standard laboratory workflow.

In this brief research report, we present and validate such a novel lysis-centrifugation protocol for laboratories equipped with Bact/ALERT^®^ (bioMérieux) and Vitek MS^®^ (bioMérieux) based on a simplified protocol first published by Foster [19].

## Materials and methods

### Routine identification of bacteria grown on solid media (standard of care, SOC)

Positive BCs were Gram stained and sub-cultured overnight on Columbia blood (bioMérieux) and chocolate agar (BD, Heidelberg, Germany) at 37°C and 5% CO_2_. The next day, a single bacterial colony was transferred from the agar plate to a single well of a Vitek^®^ MS-DS target slide (bioMérieux). One microliter of matrix (Vitek^®^ MS-CHCA, [3.1g/100μl alpha-Cyano-4-hydroxycinnamic acid]) (bioMérieux) was added and the mixture was air dried. The slide was analyzed in the Vitek^®^ MS mass spectrometer (bioMérieux, [337 nm nitrogen laser, 19.9 kV, maximum pulse rate 50 Hz, mass range 2000–20000 Da]) and only results with confidence level > 99% were used for identification. If no identification could be obtained, biochemical identification using the Vitek^®^ 2 (bioMérieux) and/or the 16S rDNA sequencing [20] were performed.

### Preparation of bacterial pellets from positive BacT/ALERT^®^ blood culture bottles

Seven hundred microliter of BC broth were mixed with 0,3 ml wash buffer 1 (0.5ml 1.5% SDS in 2.5 ml sterile distilled water) in a 1,5 ml Eppendorf tube and centrifuged for 2 min at 3,000 × *g* (Mikro 200, Hettich, Tuttlingen, Germany). The supernatant was removed, the pellet was resuspended in 1 ml of wash buffer 2 (0.5 ml 1.5% SDS in 9.5ml sterile distilled water) and the suspension was centrifuged for 2 min at 3,000 × *g*. The same procedure was then followed with wash buffer 3 (1 ml of sterile distilled water containing 1% (w/v) *N*-acetyl-l-cysteine (N-ACC)) and with 1 ml of sterile distilled water. The supernatant was removed and the remaining pellet was used for identification with the Vitek^®^ MS.

### Direct mass spectrometric identification of bacteria grown in blood bottles using the Vitek^®^ MS

One microliter of the pellet was each placed in triplicate on a Vitek^®^ MS-DS target slide (bioMérieux), 0.5 μl formic acid (Vitek^®^ MS-FA) were added to each well, and the slide was air dried. To each well 1 μl of matrix (Vitek^®^ MS-CHCA) were added. The slide was air dried and analyzed in the Vitek^®^ MS mass spectrometer (bioMérieux). A confidence level of ≥ 99% in one of the triplicates was considered an acceptable identification. No identification result was assumed if no identification was given for all triplicates. A misidentification by the RDT was assumed if it did not match the identification given by the SOC.

### Validation of the fast identification protocol using clinical samples

To assess the performance of the modified protocol, we used a convenience sample of positive BCs that were taken for routine diagnostics from patients at the University Hospital of Wuerzburg in two time periods, comprising 1250 positive BCs sampled between March 2017 and September 2017 and 1308 positive BCs between August 2018 and February 2019, respectively. Blood samples were inoculated into BacT/Alert FA (aerobic culture) (bioMérieux) and/or FN (anaerobic culture) (bioMérieux) blood culture bottles and incubated at 37°C using automated BACT/ALERT^®^ 3D system (bioMérieux) for 7 days until flagged positive. Prior inclusion to the study, Gram stain was performed and samples of the same appearance in Gram stain from the same patient within two weeks were excluded to avoid copy strains. In addition, microscopically polymicrobial samples were excluded. The resulting 266 positive samples were processed according to the SOC as outlined above. Authors had access to information that could identify individual participants during data collection.

### Statistical methods

Concordance was calculated as number of correctly identified species by RDT divided by the number of identified species by the SOC. 95%-Confidence intervals (95%-CI) were obtained by a procedure given by Clopper and Pearson [21] as implemented in the “binom.test” function of the R stats package version 4.0.3 [22].

### Ethics approval and consent to participate

This study was approved by the Ethics committee of the Medical Faculty of the University of Würzburg, Germany (reference number: 2021072801) and a waiver for informed consent was granted. All methods were performed in accordance with the relevant guidelines and regulations.

## Results

**Fig 1:**
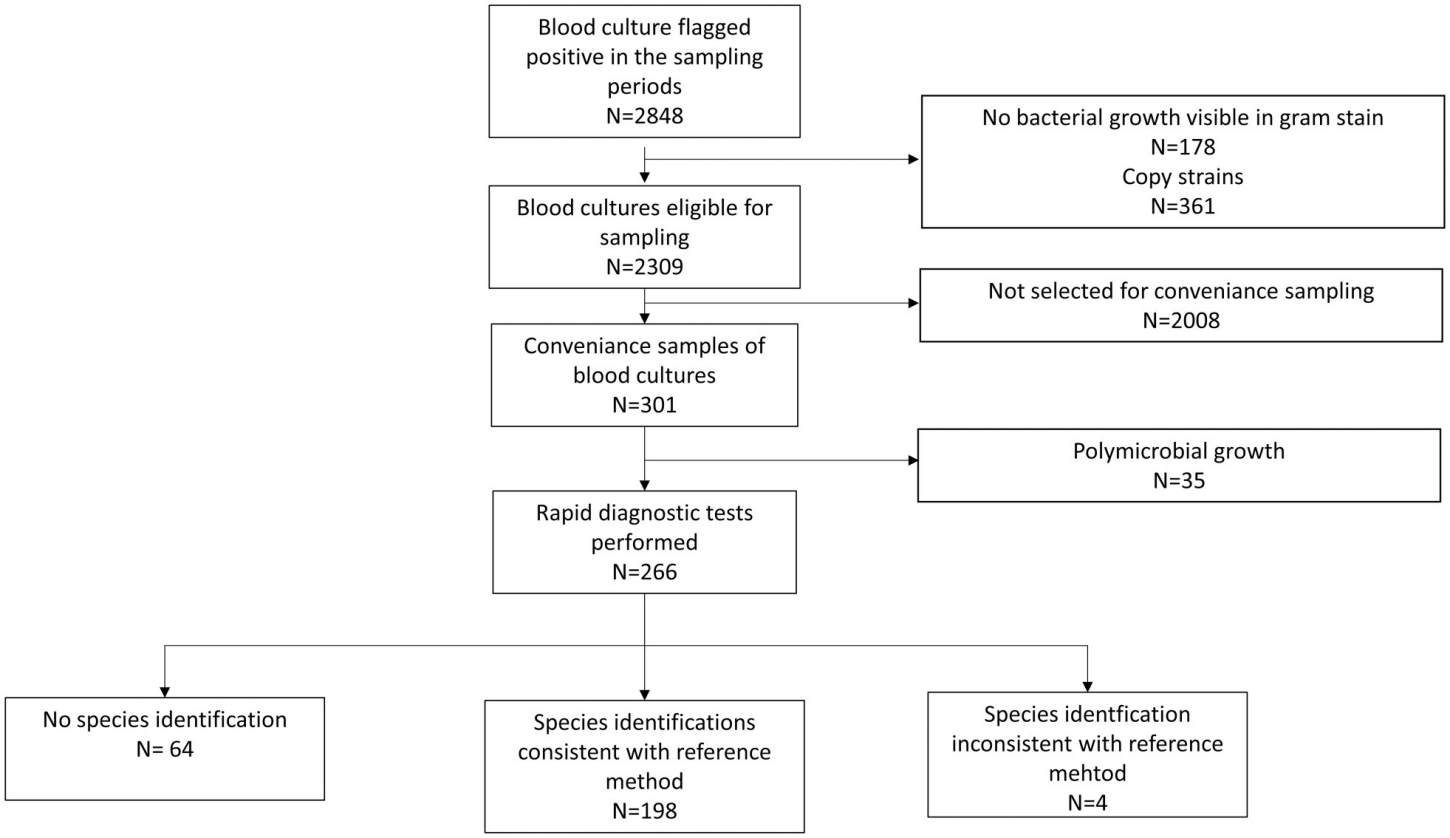
Flow diagram of the clinical blood culture samples included in the study and results of the fast protocol compared to the standard of care according to the STARD criteria [23].

After removing positive BCs with either copy strains or no visible bacterial growth in gram microscopy, 2309 positive BCs were eligible for validation (Fig 1). Of these, a convenience sample of 301 positive BCs was used, including 35 positive BCs that were excluded from further analysis due to polymicrobial growth on solid media. Bacteria identified by the SOC in the remaining 266 positive BCs (200 aerobic and 66 anaerobic) comprised 164 aerobic gram-positive bacteria, 93 aerobic gram-negative bacteria, 6 anaerobes and 3 yeast belonging to 42 species and 24 genera, respectively (Table 1). As further shown in Table 2, the distribution of the bacteria over the different groups in the convenience sample as identified by SOC was representative for all the isolates from positive BCs in the year 2017 at our laboratory (Pearson’s χ^2^ test, χ^2^ = 13.09, df = 11, p = 0.29). This suggests that there was no bias in the composition of the convenience sample with respect to a certain group of pathogen.

**Table 1:** Sensitivity and concordance of the in-house protocol on clinical samples by condition.

	Identification by reference method (n)	Any identification by rapid protocol (n)	Correct identification by rapid protocol (n)	Concordance (%)
**Gram-positive bacteria**	167	112	110	65.87%
**Staphylococci**	125	96	96	76.80%
*Staphylococcus aureus*	26	24	24	92.31%
*Staphylococcus capitis*	5	4	4	80.00%
*Staphylococcus haemolyticus*	8	6	6	75.00%
*Staphylococcus hominis*	15	13	13	86.67%
*Staphylococcus epidermidis*	67	46	46	68.66%
*Staphylococcus warneri*	2	2	2	100.00%
*Staphylococcus lugdunensis*	1	1	1	NA
*Staphylococcus saccharolyticus*	1	0	0	NA
**Streptococci**	11	2	2	18.18%
*Streptococcus parasanguinis*	1	0	0	NA
*Streptococcus anginosus*	1	0	0	NA
*Streptococcus mitis/ Streptococcus oralis*	4	1	1	25.00%
*Streptococcus pneumoniae*	3	0	0	0.00%
*Streptococcus agalactiae*	1	1	1	NA
beta- haemolytic streptococcus Group C	1	0	0	NA
**Enterococci**	18	9	7	38.89%
*Enterococcus faecalis*	9	8	6	66.67%
*Enterococcus faecium*	8	1	1	12.50%
*Enterococcus gallinarium*	1	0	0	NA
**Gram-positive anaerobes**	3	1	1	33.3%
*Brevibacterium luteolum*	1	0	0	NA
*Clostridium tertium*	1	1	1	NA
*Parvimonas micra*	1	0	0	NA
**Other Gram-positive**	10	4	4	40.0%
*Bacillus cereus group*	1	1	1	NA
*Corynebacterium glucuronolyticum*	1	0	0	NA
*Gemella haemolysans*	2	1	1	50.00%
*Gemella morbillorium*	1	0	0	NA
*Micrococcus luteus*	5	2	2	40.00%
**Gram-negative bacteria**	96	89	87	90.63%
***Enterobacterales***	80	76	75	93.75%
*Escherichia coli*	47	44	44	93.62%
*Citrobacter koseri*	3	3	3	100.00%
*Enterobacter complex*	5	4	4	80.00%
*Klebsiella oxytoca*	2	2	2	100.00%
*Klebsiella pneumoniae*	12	11	11	91.67%
*Proteus mirabilis*	7	8	7	100.00%
*Salmonella species*	2	2	2	100.00%
*Serratia liquefaciens*	1	1	1	NA
*Serratia marcescens*	1	1	1	NA
**Nonfermenter**	13	10	10	76.9%
*Pseudomonas aeruginosa*	12	10	10	83.33%
*Stenotophomonas maltophilia*	1	0	0	NA
**Gram-negative anaerobes**	3	2	2	66.6%
*Bacteroides fragilis*	1	1	1	NA
*Bacteroides thetaiotanomicron*	1	1	1	NA
*Eggerthella lenta*	1	0	0	NA
**Other**				
*Vibrio fluvialis*	0	1	0	NA
**Yeast**	3	1	1	33.33%
*Candida albicans*	1	0	0	NA
*Candida krusei*	1	1	1	NA
*Candida glabrata*	1	0	0	NA
**Total**	**266**	**202**	**198**	74.44%

**Table 2:** Frequency distribution of bacteria and yeasts in the convenience sample and in all positive blood cultures for the reference year 2017.

Group	Convenience sample	Total 2017
Potential contaminants (skin or environmental)^(1)^	109	1025
*Staphylococcus aureus*	26	215
α-haemolytic streptococci	6	94
β-haemolytic streptococci	2	33
*Streptococcus pneumonia*	3	22
*Enterococcus* spp.	18	171
*Enterobacterales*	80	585
*Pseudomonas aeruginosa*	12	74
Non-fermenters (excl. *P*. *aeruginosa*)	1	19
Anaerobes (excl. *Propionibacterium* spp.)	6	39
Yeasts	3	76
Others	0	11
Total	266	2364

^(1)^ Coagulase-negative staphylococci, spore-forming aerobes except *Bacillus anthracis*, non-sporeforming rods except *Listeria* spec. and *Corynebacterium diphtheriae*, *Brevibacterium* spec., *Micrococcus* spec., *Propionibacterium* (*Cutibacterium*) spec.

On the species level concordant results between the SOC and the rapid identification protocol were obtained for 198 positive BCs (74.4%, 95%-CI = [68.8%, 79.6%]) (Fig 1 and Table 1). The rapid identification protocol failed to provide a species identification in 64 and gave a wrong species identification in only 4 BCs, respectively. It was thus correct for 98.0% (95-CI = [95.0%, 99.5%]) of the samples with an identification and in turn failed to give an identification in 24.1% of the tested clinical samples (95-CI = [19.1%, 29.7%]).

One-hundred ten of the 167 BCs with gram-positive bacteria identified by the SOC were correctly identified at the species level also by the rapid identification protocol (65.9%, 95%-CI = [58.1%, 73.0%]). There was however a significant heterogeneity of the concordance values between the SOC and the rapid identification protocol among the gram-positive bacteria at the genus level (χ^2^ = 24.1, df = 2, p-value < 0.00001, 3-sample test for equality of proportions without continuity correction), ranging from 76.8% (95%-CI = [68.4%, 83.9%]) for staphylococci to 18.2% (95%-CI = [2.2%, 51.8%]) for streptococci. Of the 26 *Staphylococcus aureus* isolates 24 were correctly identified to the species level by the rapid identification protocol (92.3%, 95%-CI = [74.9%, 99.1%]) and 72 of the 99 coagulase-negative *Staphylococcus* (72.7%, 95%-CI = [62.9%, 81.2%]). Among the enterococci the rapid identification protocol successfully identified 6 of 9 *Enterococcus faecalis* to the species level but only 1 of 8 *E*. *faecium*.

Of the 96 gram-negative pathogens detected in the BCs by the SOC, 87 were correctly identified by the rapid identification protocol (91.4%, 95%-CI = [83.8%, 93.2%]) with an excellent identification rate of 75 out of 80 for *Enterobacterales* (93.7%, 95%-CI = [86.0%, 97.9%]). Ten of the 13 non-fermenting Gram-negative rods identified by the SOC were also correctly identified by the rapid identification protocol (76.9%, 95%-CI = [46.2%, 95.0%]), which was not significantly different yet from the respective value for the *Enterobacterales* (χ^2^ = 2.17, df = 1, p > 0.1,).

For anaerobes correct species identification was achieved in 3 of 6 samples and in 1 of 3 samples for yeasts.

Four pathogens were misidentified (all with a confidence level of 99.9%): One *Streptococcus pneumoniae* and one *Streptococccus mitis/oralis* were both misidentified as *E*. *faecalis*, one *Parvimonas micra* was misidentified as *Vibrio fluvialis* and one *Escherichia coli* was misidentified as *Proteus mirabilis*.

## Discussion

The present study evaluates the accuracy of the Vitek^®^ MS for the direct identification of bacteria from positive BacT/Alert^®^ BC bottles using a simplified version of a protocol first described by Foster [19]. The main aim was to reduce additional equipment and hands-on time to a minimum to allow easy integration into the routine laboratory work-flow at minimal extra cost. By making only a single attempt to identify the microorganism and by reducing the sample volume given in the original protocol[19] we could reduce the hands-on time for sample preparation and measurement from 30–45 min to about 15 min for even up to five samples. This is shorter than for most other protocols using a similar lysis-centrifugation approach for the combination of BacT/Alert^®^ BC bottles and the Vitek^®^ MS mass spectrometer [16,24–29].

The estimated costs per sample were 0.65 € for pellet extraction plus 1.15€ for the VitekMS^®^ consumables, adding up to approx. 1.80€ per sample which is much cheaper than, e.g., the commercial Vitek^®^ MS Blood Culture Kit (bioMérieux) with about 8€ per sample.

Despite its lower costs, the overall performance of our in-house protocol was yet comparable to the commercial Vitek^®^ MS Blood Culture Kit (bioMérieux) with a reported 77.8% correct identification rate at the species level (95%-CI = [72.2%, 82.7%]) [27].

In line with previous studies using a similar approach [16,24–30] it showed in particular excellent performance for gram-negative bacteria, in particular *Enterobacterales*, *P*. *aeruginos* and *S*. *aureus* but failed so for streptococci and enterococci.

Previous studies using the combination of BacT/Alert^®^ BC bottles and the Vitek^®^ MS mass spectrometer consistently reported a higher rate of correct identifications for gram-negative bacteria (average 90.4%, 95%-CI = [76.5%, 98.5%]) than for gram-positive bacteria (74.9%, 95%-CI = [53.2%, 95.0%]) [16,24–30]. The thicker peptidoglycan layer of the gram-positive cell wall renders these bacteria more resistant to cleavage. Degrading of bacterial cell wall is an essential preparation step to achieve valid spectra by mass spectrometry. Therefore, optimized protocols for species identification of gram-positive bacteria using lysozyme or formic acid have been proposed for conventional mass spectrometry [31,32]. Albeit rapid detection protocols vary considerably, they all make no difference in the treatment of gram-positive and gram-negative bacteria. Given the altered composition of the cell walls of these bacteria, accounting for such difference is thus likely to result in further improvements yet.

During the course of this study two similar lysis-centrifugation protocols for the combination of BacT/Alert^®^ BCs with the Vitek^®^ MS of were published that showed overall identification rates of 83.9% (95%-CI = [80.6%, 86.9%]) [29] and 84.9% (95%-CI = [80.5%, 88.7%]) [30], respectively. They differ from the presented method mainly in the use of different detergents used for protein extraction (Triton (30) and saponin (29) respectively, instead of SDS), indicating that optimization of the extraction chemistry is likely to further improve the performance of rapid identification methods.

## Limitations

Our study has several limitations. First, for direct ID from positive BC bottles, poly-microbial infections were excluded and, second, due to its relatively small size, only a limited number of some important organisms such as in particular yeasts and anaerobes were included. Third, we tested convenience samples as work force availability in the routine diagnostic laboratory allowed for. However, positive BC bottles were tested in a routine diagnostic setting and the spectrum of organisms tested is representative of the mixture of BC isolates at our center. Last, our SOC was considered as a benchmark to study the performance of the optimized protocol. The SOC is continuously monitored by internal and external quality assessment (DAkkS). Therefore, we consider it unlikely that generated false bacterial species identification. However, we clearly cannot exclude misidentifications by the SOC.

## Abbreviations

BC: blood culture
BSI: bloodstream infections
CHCA: alpha-Cyano-4-hydroxycinnamic acid
CI: confidence interval
DAkkS: Deutsche Akkreditierungsstelle GmbH (German Accreditation Body)
DNA: deoxyribonucleic acid
MS: mass spectrometry
rDNA: ribosomal deoxyribonucleic acid
RDT: rapid diagnostic test
*SOC*: standard of care
